# Classification-based pathway analysis using GPNet with novel *P*-value computation

**DOI:** 10.1093/bib/bbaf039

**Published:** 2025-01-29

**Authors:** Hao Lu, Mostafa Rezapour, Haseebullah Baha, Muhammad Khalid Khan Niazi, Aarthi Narayanan, Metin Nafi Gurcan

**Affiliations:** Center for Artificial Intelligence Research, Wake Forest University School of Medicine, Winston-Salem, NC 27101, United States; Center for Artificial Intelligence Research, Wake Forest University School of Medicine, Winston-Salem, NC 27101, United States; School of Systems Biology, College of Science, George Mason University, Fairfax, VA 22030, United States; Department of Pathology, Ohio State University, 2255 Kenny Rd, Columbus, OH 43210, United States; Department of Biology, George Mason University, Fairfax, VA 22030, United States; Center for Artificial Intelligence Research, Wake Forest University School of Medicine, Winston-Salem, NC 27101, United States

**Keywords:** pathway analysis, Gene PointNet, deep learning, bioinformatics, gene interactions, *P*-value computation

## Abstract

Pathway analysis plays a critical role in bioinformatics, enabling researchers to identify biological pathways associated with various conditions by analyzing gene expression data. However, the rise of large, multi-center datasets has highlighted limitations in traditional methods like Over-Representation Analysis (ORA) and Functional Class Scoring (FCS), which struggle with low signal-to-noise ratios (SNR) and large sample sizes. To tackle these challenges, we use a deep learning-based classification method, Gene PointNet, and a novel $P$-value computation approach leveraging the confusion matrix to address pathway analysis tasks. We validated our method effectiveness through a comparative study using a simulated dataset and RNA-Seq data from The Cancer Genome Atlas breast cancer dataset. Our method was benchmarked against traditional techniques (ORA, FCS), shallow machine learning models (logistic regression, support vector machine), and deep learning approaches (DeepHisCom, PASNet). The results demonstrate that GPNet outperforms these methods in low-SNR, large-sample datasets, where it remains robust and reliable, significantly reducing both Type I error and improving power. This makes our method well suited for pathway analysis in large, multi-center studies. The code can be found at https://github.com/haolu123/GPNet_pathway">https://github.com/haolu123/GPNet_pathway

## Introduction

Pathway enrichment analysis (PEA) has traditionally relied on hypothesis-testing approaches, such as Functional Class Scoring (FCS) [1] and Over-Representation Analysis (ORA) [2], and Topology-based methods [3, 4]. However, these hypothesis testing approaches are based on predefined, rigid, and highly constrained statistical models. This rigid framework prevents the models from being updated or adapted as new data becomes available, which means that increasing the dataset size does not significantly improve their performance. The variance introduced by differing experimental conditions across datasets is challenging to model using traditional statistical methods. Consequently, their performance declines when applied to large, high noise and variability datasets.

In recent years, the amount of genomic data being generated has surged, with large-scale datasets released by various research centers [5]. These datasets, while valuable, come with challenges. Differences in experimental conditions across studies introduce significant noise, even after normalization, leading to high variability in the data and a reduced signal-to-noise ratio (SNR) [6, 7]. This situation presents a substantial challenge for traditional hypothesis-testing methods, which were originally designed for small sample sizes and struggle to maintain reliability when faced with noisy, large datasets (see Section 5). Thus, there is a growing need for new PEA methods that can effectively handle large datasets and the associated noise.

Deep learning, with its ability to model complex relationships and work efficiently with large datasets, has emerged as a powerful tool in bioinformatics [8–10]. Classification-based deep learning methods are particularly well suited for addressing the challenges posed by low SNR and large genomic datasets, as they can detect subtle differences in gene expression [9–11]. One such approach is DeepHisCoM [10], which employs deep learning for pathway analysis. However, it requires permutation tests to compute $P$-values, which can be computationally expensive and inefficient. To overcome this limitation, we propose a novel $P$-value computation method leveraging the classification confusion matrix. This approach eliminates the need for permutation tests, significantly improving computational efficiency. Additionally, we leverage GPNet [11], a deep learning model we previously introduced, to model the complex gene interactions within pathways, providing a more accurate and scalable solution for PEA in large datasets.

To validate the effectiveness of our method, we conducted extensive experiments on two datasets. The first is a simulated dataset generated according to established principles [12], allowing us to evaluate our approach under controlled conditions systematically. The second dataset consists of RNA-Seq data from The Cancer Genome Atlas (TCGA) breast cancer dataset, which we manually enhanced to establish ground truth [13]. The details of these to dataset are explained in Section 4. We compared the performance of our method against traditional hypothesis-testing approaches: FCS and ORA; other linear classification-based methods: logistic regression (LR) [14], support vector machines (SVM) [15]; and other deep learning based methods: PASNet [16] DeepHisCoM [10]. As demonstrated in Section 5, our method consistently outperforms traditional techniques, especially in scenarios where small changes in expression levels need to be detected in pathways within large sample sizes. The main contributions of this paper can be summarized in two aspects:

- We modify the structure and training process of GPNet to adapt it to PEA tasks while retaining the knowledge acquired during pretraining
- We proposed a novel method for $P$-value computation based on the classification confusion matrix is proposed. This method eliminates the need for computationally expensive permutation tests, improving efficiency without sacrificing accuracy.

The paper is organized as follows. In Section 2, we introduce the related works on traditional PEA and deep learning evaluation. In Section 3, we present our proposed GPNet and $P$-value computing methods. Considering the complexity of evaluating data generalization, we describe how to generalize the two evaluation datasets in Section 4. In Section 5, we present our experiments and results. Finally, our discussion is provided in Section 6.

## Related works

### Traditional methods for PEA

PEA is a key bioinformatics method for understanding gene expression data. Traditional PEA methods include ORA [2, 17, 18], FCS [1, 19] methods, and topology-based methods [3, 4].

- ORA: ORA [2, 17, 18] identifies whether a predefined gene set (e.g. a pathway) is over-represented among differentially expressed genes using statistical tests like the hypergeometric or Fisher’s exact test. Though simple and easy to use, ORA classifies genes into significant and non-significant categories, which may cause information loss and overlooks gene interactions within pathways.
- FCS: FCS methods, like GSEA [1, 19], assess gene sets based on expression levels across the entire dataset without thresholds, minimizing information loss. However, they can be computationally intensive and often assume gene independence, which may not reflect real biological interactions.
- Topology-based methods: these methods [3, 4] use pathway network structures to account for gene interactions, providing more biologically relevant insights. However, they require detailed pathway information, which may not be available for all genes or species. In contrast, deep learning methods can learn gene interactions directly from data, without needing predefined pathway structures.

### Deep learning methods for PEA

Deep learning has introduced new opportunities for PEA by modeling complex, non-linear relationships in gene expression data [20].

- DeepHisCoM: this method [10] combines hierarchical community detection with deep learning to improve pathway enrichment sensitivity. However, it still relies on permutation tests to compute $P$-values, which can be computationally expensive.
- PASNet: PASNet [16] integrates pathway information with autoencoder architectures to enhance feature extraction, improving pathway identification by combining prior biological knowledge with deep learning’s representation capabilities.

Other notable deep learning approaches include Pathway Deep Learning [21], and gene2vec [21] use CNNs and embedding techniques, respectively, to model gene interactions. While promising, these methods often require significant computational resources and do not easily provide $P$-values, limiting their widespread use.

### PointNet for PEA

GPNet builds on the principles of PointNet [22], a method originally developed for 3D point cloud recognition. We adapted this architecture to handle gene expression data, treating genes as points characterized by their expression levels and interactions. GPNet [11] effectively captures complex within-pathway interactions and is particularly suited for large, noisy datasets from multiple sources.

We also introduce a novel $P$-value computation method using the classification confusion matrix, eliminating the need for costly permutation tests. This makes GPNet an efficient and scalable solution for PEA, offering improved accuracy and reduced computational burden in large-scale studies.

## Methods

In this paper, we focus on case-control studies, which identify gene sets that are differentially expressed between cases (e.g. diseased) and controls (e.g. healthy). Throughout this paper, we will refer to cases and controls as class 1 and class 2. The main idea behind this method is that if a gene set shows differential expression in a certain disease condition, then the expression data of that gene set should be able to effectively classify disease and healthy samples. In contrast, if there is no differential expression, the classification should be less effective. Therefore, the core of this method is to train a deep learning classifier that can take the expression data of any gene set as input and output the classification of samples (healthy or diseased). Based on the classifier accuracy, measured by the confusion matrix, we estimate the confidence ($P$-value) of whether the gene set exhibits differential expression.

### Structure of GPNet

**Figure 1:**
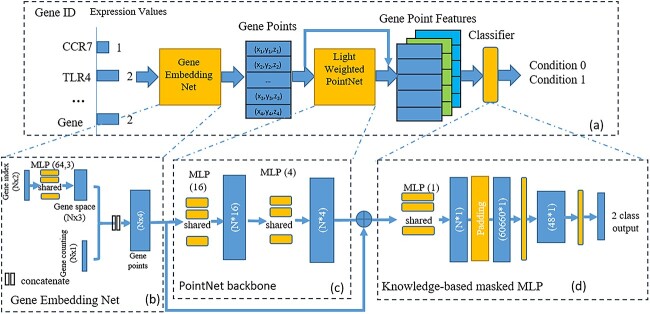
Schematic diagram of the Gene PointNet end-to-end training process, where N is the input gene number. (a) Overview of the Gene PointNet architecture, (b) structure of the gene embedding network, which converts gene expression data into point cloud format, (c) structure of the PointNet backbone, which extracts features from the point cloud data, (d) structure of the knowledge-based MLP classifier, which uses point cloud features to predict class categories, and (e) detailed structure of the normalization network and token/feature transformation network used in GPNet.

In this work, we use our preproposed GPNet [11] as our deep classification model. This model has shown good classification performance in RNA-Seq data. Figure 1 illustrate the frame work of GPNet. It contains 3 key parts: (1) Gene Point Cloud Embedding, illustrate as Fig. 1(b), which transforms gene expression data into a high-dimensional point cloud, (2) PointNet backbone, illustrated as Fig. 1(c), which is used to process the high-dimensional point cloud data, and (3) Knowledge-based MLP Classifier, illustrate as Fig. 1(d), which is a classification header to predict the class category by integrating the knowledge of the biological pathway. Compared to the GPNet structure described in reference [11], this paper introduces three key modifications to the GPNet structure:

- Removal of all TNet modules: all TNet modules have been removed, as reference [11] demonstrated that these modules provide limited performance improvement.
- Addition of residual connections in the PointNet backbone: residual connections have been incorporated into the PointNet backbone, enabling faster and more stable convergence of GPNet.
- Zero-padding in the knowledge-based MLP module: zero-padding has been introduced in the knowledge-based MLP module, allowing inputs of arbitrary dimensions to share the pretrained GPNet network parameters. This enhances the convenience of transfer learning.

For a gene expression sample with $N$ genes, let $\mathbf{x}=[x_{1},x_{2},...,x_{N}]$ represent the expression counts, and $\mathbf{g}=[g_{1},g_{2},...,g_{N}]$ denote the corresponding gene names. Following the approach in [11], we assign each gene name a unique integer pair as its gene token, and then go through a 1D convolution layer to get gene embedding. The expression counts $x$ are normalized using Z-score normalization. The embeddings of the gene points are the concatenation of normalized counts and gene embeddings, which can be expressed as


(1) \begin{align*} \mathbf{h} = \begin{bmatrix} \hat{\mathbf{x}} \\ \text{emb}_{g}\mathbf{t}\end{bmatrix},\end{align*}

where $\hat{\mathbf{x}}=\text{Norm}{\mathbf{x}}$, $\mathbf{t}=[id(g_{1}),id(g_{2}),...id(g_{N})]$, and $\text{emb}_{g}$ is $\text{Conv1D}(2\rightarrow 3)$ operation.

After simplification, the PointNet backbone is effectively reduced to two 1D convolution layers. As a result, the extracted gene features can be expressed as follows:


(2) \begin{align*} \mathbf{f}=\text{Conv1D}_{2}(\text{Conv1D}_{1}(\mathbf{h})),\end{align*}

$\mathbf{f}\in \mathcal{R}^{N\times 4}$ represents the gene feature matrix, the output from PointNet backbone. Then $\mathbf{f}$ will go through a 1D convolution layer to map the feature matrix to a $N\times 1$ vectors. This layer can be treated as a channel-wise weighted average pooling, corresponding to the global average pooling layer in original PointNet [22]. The original GPNet [11] was trained on data with 60 660 genes, which contains comprehensive gene annotations and alternative transcripts. To accommodate input with any number of genes, we introduced zero-padding in the Knowledge-based masked MLP. A vector of length $N$ is padded to a $60\,660times 1$ vector before being processed by the masked MLP. This process can be represented as follows:


(3) \begin{align*} \mathbf{S} = MLP(Pad(\mathbf{f})), \end{align*}

(4) \begin{align*} \mathbf{O} = \text{Linear}(\text{ReLU}(\mathbf{W}\otimes \mathbf{M}\mathbf{S}^{T}+\mathbf{B})), \end{align*}

where $\mathbf{S}\in \mathcal{R}^{N\times 1}$ represents the 1D feature vector, $\mathbf{W}$ and $\mathbf{M}$ denote the linear transformation weights and mask matrix, respectively, described in [11], and $\mathbf{O}$ is the binary output.

### GPNet training procedure for PEA

GPNet [11] was originally designed for tumor classification using full gene expression data. To adapt GPNet for PEA, some modifications to the training procedure are required. Specifically, we proposed a method that trains only a small subset of bias weights while keeping the pre-trained parameters of GPNet unchanged.

As Equation 4, $\mathbf{B}$ is the bias vector. we reinitialized the bias parameters $\mathbf{B}$ in the Knowledge-based MLP layers using Kaiming initialization [23]. and only updated $\mathbf{B}$ during the fine-tuning. This approach adapts the model to specific pathways without altering previously trained weights, minimizing computational costs and reducing the risk of overfitting.

Importantly, because the original GPNet [11] had already been trained on the TCGA BRCA dataset, to ensure fairness, all results presented in this study were obtained by training the model from scratch.

### $P$ -value computation from confusion matrix

To assess the statistical significance of the pathway-specific predictions, we compute $P$-values using the confusion matrix from the classification results. For a binary classification problem, the confusion matrix is structured as follows:

-
$a_{11}$ : true positives (class 1 correctly predicted as class 1)
-
$a_{12}$ : false negatives (class 1 incorrectly predicted as class 2)
-
$a_{21}$ : false positives (class 2 incorrectly predicted as class 1)
-
$a_{22}$ : True negatives (class 2 correctly predicted as class 2)

The core idea is to evaluate whether the predictions for class 1 and class 2 are statistically better than random guessing. This is done by treating the classification task as a series of “trials” and using the binomial distribution to model the number of correct predictions.

For class 1, the binomial distribution models the number of correct predictions (successes) out of a fixed number of trials (total samples in class 1, $n_{1}=a_{11}+a_{12}$) with a given probability of success ($p_{1}$, the proportion of class 1 samples in the dataset).

**Prior Probability Calculation** For class 1, the prior probability, $p_{1}$, is calculated as follows:


(5) \begin{align*}& p_{1} = \frac{a_{11}+a_{12}}{a_{11}+a_{12}+a_{21}+a_{22}}\end{align*}

This represents the proportion of samples that actually belong to class 1. Similarly, the prior probability for class 2, $p_{2}$, is


(6) \begin{align*}& p_{2} = \frac{a_{21}+a_{22}}{a_{11}+a_{12}+a_{21}+a_{22}}\end{align*}

**$\boldsymbol{P}$ -value computation for class 1** To compute the $P$-value for class 1, we model the number of correct predictions using the binomial distribution $B(n_{1},p_{1})$,where $n_{1}=a_{11}+a_{12}$ is the total number of samples in class 1. The $P$-value, $P_{value1}$, represents the probability that a random prediction would have fewer errors (false negatives) than the actual model:


(7) \begin{align*}& P_{value1} = \mathit{CDF}(k=a_{12},n=a_{11}+a_{12},p=1-p_{1}),\end{align*}

Here, the CDF computes the probability of observing up to $k=a_{12}$ errors (false negatives) under the binomial distribution.

**$\boldsymbol{P}$ -value computation for class 2** Similarly, the $P$-value for class 2, $P_{value2}$, is computed using the binomial distribution $B(n_{2},p_{2})$, where $n_{2}=a_{21}+a_{22}$. The $P$-value for class 2 is


(8) \begin{align*}& P_{value2} = \mathit{CDF}(k=a_{22},n=a_{21}+a_{22},p=1-p_{2}),\end{align*}

This $P$-value represents the likelihood that a random model would have fewer errors for class 2 than the actual model.

**Overall $\boldsymbol{P}$-value computation** To compute a single $P$-value for the overall classification performance, we combine the $P$-values from both classes. Let the random prediction accuracy for class 1 and class 2 be $Acc_{r1}$ and $Acc_{r2}$, and the actual model accuracy for class 1 and class 2 be $Acc_{g1}$ and $Acc_{g2}$. The overall model accuracy is defined as follows:


(9) \begin{align*}& Acc_{g} = \frac{a_{11}+a_{22}}{a_{11}+a_{12}+a_{21}+a_{22}}\end{align*}

The combined $P$-value, $P_{v}alue$, reflects the probability that a random model would perform as well as or better than the actual model. We calculate this as follows:


(10) \begin{align*}& P_{value}=1-(1-P_{value1})(1-P_{value2})\end{align*}

Here, $P_{value1}$ and $P_{value2}$ are the $P$-values for class 1 and class 2, respectively. This combined $P$-value provides a robust measure of the statistical significance of the model’s pathway-specific predictions, reflecting its overall accuracy in predicting both classes.

## Data generation for PEA

One of the most challenging aspects of evaluating the performance of PEA algorithms is determining the ground truth of pathway expression. To address this, we generated two datasets, following methodologies outlined in [12] for a purely simulated dataset and [13] for real data with manually added stimulation. These datasets provide a known ground truth for evaluation.

### Pure simulated dataset

We generated a simulated dataset following the method in [12], with some modifications to focus on sample size and SNR effects on power and Type I error. Both the control and treatment groups contain 250 samples each, with gene expression data drawn from a multivariate normal distribution. The dataset consists of 10 000 genes, divided into 50 non-overlapping gene sets, with 500 genes in each set.

A generated 10 000$\times $10 000 covariance matrix was used, with an average correlation of 0.8 among genes within the same pathway and 0.05 between genes in different pathways. Five randomly selected gene sets exhibit differential expression, where the changes are introduced by modifying the mean vector of the treatment samples. The magnitude of expression differences between the control and treatment groups is controlled by adjusting the SNR, computed as $SNR=\frac{{mean}^{2}}{sigma^{2}}$. This allows us to model varying intensities of gene expression changes, creating conditions for a comprehensive evaluation of the PEA algorithms’ performance in terms of power and Type I error.

### Real data with manually added stimulation

To generate the real data with manually added stimulation, we follow the experimental design outlined in the paper [13].For the real dataset, we used a breast cancer gene expression study (BRCA) from TCGA. We focused on 114 signaling and metabolic pathways from the Kyoto Encyclopedia of Genes and Genomes ([24]), and expression data from 2784 genes that have matched Entrez IDs. The data set consists of 1078 samples in total, 286 estrogen-receptor-negative (ER−) and 792 estrogen-receptor-positive (ER+). To consist with the simulated data, we randomly select 250 samples from ER− and ER+ in this study. The original RNA-Seq data were obtained from the Genomic Data Commons (GDC) portal [25]. To remove potential biases and ensure balanced variance between the two groups, we permuted the sample labels (estrogen-receptor-negative and estrogen-receptor-positive). This step equalizes the variance between the control and treatment groups, preventing artifacts that could skew the analysis.

Key genes within pathways were identified using the betweenness centrality method, which ranks genes based on their network centrality scores. Genes with the highest centrality were considered to be differentially expressed. To introduce realistic expression changes, we manually modified the mean expression values of these key genes in selected pathways, simulating different levels of expression intensity. This controlled modification creates a gradient of stimulation, which is crucial for evaluating the sensitivity and specificity of PEA algorithms.

By generating both simulated and real datasets with known ground truth, we ensure robust and realistic testing of pathway enrichment algorithms. These datasets provide a controlled environment to measure the algorithms’ power and Type I error, offering a comprehensive framework for evaluating the efficacy of different PEA methods under both ideal and real-world conditions.

## Results and discussion

To comprehensively assess the performance of our proposed GPNet algorithm, we compared it against both traditional PEA methods and state-of-the-art (SOTA) deep learning approaches. We selected GSEA [1] and ORA [2] as traditional PEA methods due to their widespread use and robust performance across diverse types of data. We also included PASNet [16] and DeepHisCom [10] as the SOTA deep learning methods for PEA. These models have been widely recognized for leveraging deep learning architectures to improve the sensitivity and accuracy of pathway analysis. Additionally, to evaluate the difference between deep learning and traditional machine learning, we replaced the GPNet model with shallow machine learning methods, including LR [14] and SVM [15]. LR and SVM are commonly used linear models that perform well on simpler datasets and are often used as a baseline when comparing the performance of deep learning models. The evaluation focused on Type I error rates and statistical power across various conditions, with each experiment conducted over 100 simulation replications to ensure robustness and reliability of the results.

LR, SVM, PASNet, and GPNet are used method described in section 3.3 to compute $P$-values. DeepHisCom is used permutation tests as [10] to compute $P$-values.

We evaluated the PEA results using two datasets: a purely simulated dataset and a real-world dataset from TCGA. For the simulated data, both the control and treatment groups contained 250 samples, with expression data generated for 10 000 genes. These genes were organized into 20 non-overlapping pathways. In each simulation, five pathways were randomly selected, and within each selected pathway, the top 10% of genes with the highest betweenness centrality scores were designated as differentially expressed. For the real-world data, we used the TCGA breast cancer RNA-Seq dataset, downloaded from the GDC, which consists of 250 control and 250 treatment samples. The dataset contains expression data for 39 376 genes. For evaluation, 11 cancer-related pathways were selected: Central carbon metabolism in cancer, Choline metabolism in cancer, Sphingolipid signaling pathway, mTOR signaling pathway, Adrenergic signaling in cardiomyocytes, VEGF signaling pathway, Apelin signaling pathway, TNF signaling pathway, Retrograde endocannabinoid signaling, GnRH signaling pathway, and Oxytocin signaling pathway. In each simulation, one to three pathways were randomly selected. From these selected pathways, we identified genes unique to each pathway, and the top 10% of genes with the highest betweenness centrality scores were chosen to exhibit differential expression.

### Efficient comparison

In this section, we demonstrate the computational efficiency of our proposed method compared to (SOTA) deep learning approaches. Specifically, we compute the floating-point operations (FLOPs) only during the inference process, which includes a forward prediction process and a single $P$-value computation for PASNet and GPNet using our proposed $P$-value computational method. For DeepHisCom, the inference process contains $1000$ times permutation tests.

We compared our GPNet model with two other deep learning methods: DeepHisCom [10] and PASNet [16]. In the DeepHisCom study, the authors performed a permutation test 100 000 times to obtain reliable $P$-values. While this approach enhances the reliability of their results, it is extremely resource-intensive. For instance, replicating their approach in our setting would require over 300 days of computation due to the limited computational resources at our disposal. Given these constraints, we reduced the number of permutations to 1000 in our experiments, which was a more feasible approach within our resources. We acknowledge that this reduction may impact the performance of DeepHisCom, particularly in Type I error rates and statistical power tests. However, from a computational efficiency perspective, this adjustment allows for a fair comparison between the methods. The computational efficiency comparison is summarized in Table 1, measured by the number of FLOPs. As shown, GPNet (our method) requires significantly fewer FLOPs (1.01G) compared to both DeepHistCom (32.36G) and PASNet (5.32G). This highlights the computational advantage of GPNet, making it a more resource-efficient choice, especially when computational resources are limited.

**Table 1:** Computational efficiency comparison (FLOPs) between our method (GPNet) and state-of-the-art deep learning methods (lower FLOPs indicate higher efficiency)

Method	FLOPs
DeepHisCom [10]	32.36G
PASNet [16]	5.32G
**GPNet (OURS)**	**1.01G**

### PEA evaluation

**Figure 2:**
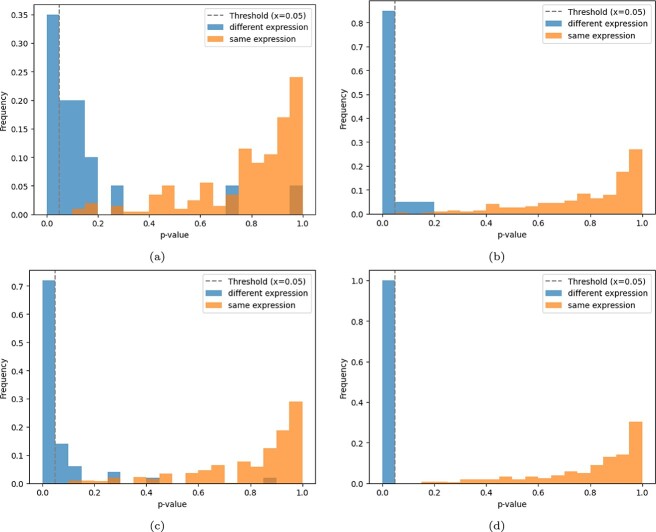
$P$ -value distributions for real and simulated datasets under different sample sizes. (a) $P$-value distribution computed by GPNet for the real dataset with a sample size of 100. (b) $P$-value distribution computed by GPNet for the real dataset with a sample size of 250. (c) $P$-value distribution computed by GPNet for the simulated dataset with a sample size of 100. (d) $P$-value distribution computed by GPNet for the simulated dataset with a sample size of 250.

**$P$ -value evaluation** Figure 2 illustrates the $P$-value distribution for both real and simulated data under a SNR of 0.05, with sample sizes of 100 and 250 for the control and treatment groups. From the figure, it is evident that for the differentially expressed groups, the $P$-values are consistently smaller, and as the sample size increases, a larger proportion of these $P$-values fall below the 0.05 threshold. Conversely, for the groups without differential expression, the majority of $P$-values remain above 0.05. This pattern demonstrates that our method provides a reliable and effective $P$-value evaluation, particularly as sample size increases, enabling it to better differentiate between expressed and non-expressed groups.

### Type I error and power evaluation

In this section, we evaluate the Type I error rate and power of our proposed method, GPNet, alongside other PEA methods.

The Type I error rate was assessed by setting a threshold to ensure that the Type II error rate (false negative rate) remained below 0.1. This constraint allowed us to fairly evaluate how prone each method was to falsely identify non-existent pathway enrichments.

Power is defined as a method’s ability to correctly identify true pathway enrichments, was evaluated by setting a threshold to ensure that the Type I error rate remained below 0.1. This ensured a fair comparison by controlling false positive rates while assessing each method’s sensitivity to detect true signals.

**Figure 3:**
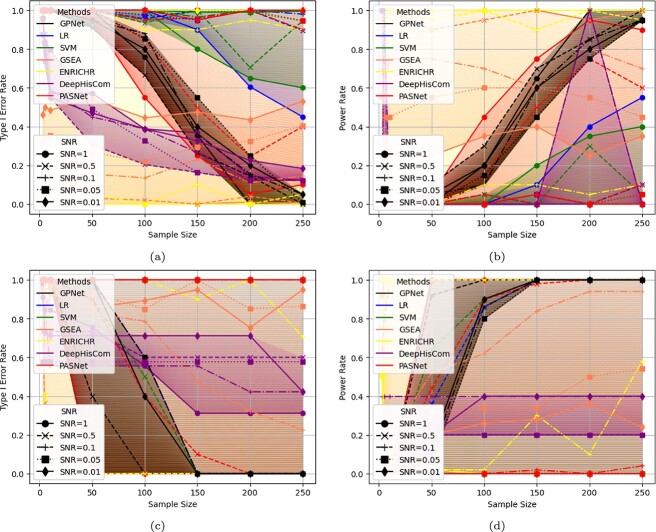
Type I error and power for different Pathway Enrichment Analysis (PEA) methods against varying sample sizes for both real and simulated datasets with different Signal-to-Noise Ratios (SNRs). (a) Type I error rates using various PEA methods on a real dataset, (b) statistical power of various PEA methods on a real dataset, (c) Type I error rates using various PEA methods on a simulated dataset, (d) statistical power of various PEA methods on a simulated dataset. Methods compared include our proposed GPNet, logistic regression, SVM, GSEA, ORA, DeepHisCom, and PASNet.

We used both real and simulated datasets to compare the Type I error rates and power of GPNet against several other methods, including GSEA, ORA, SVM, LR, DeepHisCom, and PASNet. Each test was replicated 100 times to ensure statistical reliability. Figure 3 presents the Type I error and power of these methods under varying sample sizes and SNR for both real and simulated datasets. The results for different methods are color-coded: black for GPNet (ours), blue for LR, green for SVM, coral for GSEA, yellow for ORA, purple for DeepHisCom, and red for PASNet.

From Fig. 3, we can derive the following insights:

- Shallow machine learning methods (LR and SVM): these methods are not suitable for PEA tasks, as they perform poorly across all SNR levels and sample sizes for both real and simulated datasets.
- Traditional PEA methods (GSEA and ORA): these methods perform well in cases of high SNR and small sample sizes. However, their performance is highly sensitive to changes in SNR, and increasing the sample size does not improve their results. This limits their ability to handle data from multi-center studies where larger datasets are collected, but differences in data collection environments lead to reduced SNR.
- DeepHisCom: while DeepHisCom is robust to SNR changes and improves with larger sample sizes, it struggles to reduce Type I error below 0.1, regardless of how much the sample size increases. As shown in Fig. 3(b), we often cannot report power results under the 0.1 Type I error threshold. This limitation may be due to the fact that we performed only 1000 permutation tests, compared to the 100 000 permutations used in the original paper. However, such a large computational cost is infeasible for our resources.
- PASNet: while PASNet improves with larger sample sizes, it is highly sensitive to changes in SNR. When SNR decreases, increasing the sample size has little effect on improving performance, limiting its utility in low SNR scenarios.
- GPNet (Ours): our proposed method, GPNet remaining robust to changes in SNR. As the sample size increases, both the Type I error approaches 0 and the power approaches 1, indicating that GPNet reliably delivers accurate results as long as sufficient sample size is available. This suggests that GPNet is well suited for large-scale datasets from multi-center studies, where it can handle increased noise while maintaining performance.

In sum, while GPNet may not perform optimally with small sample sizes, it consistently delivers reliable results when sufficient data is available. Its robustness to noise makes it particularly well suited for complex, large-scale, multi-center datasets, ensuring dependable PEA even in challenging conditions.

## Conclusions

In this study, we introduced the usage of GPNet for PEA and a method to compute $P$-value using confusion matrix. We compared our method with traditional PEA methods (GSEA and ORA), linear machine learning models (LR and SVM) and other deep learning methods (DeepHisCom and PASNet). Our findings demonstrate that GPNet significantly outperforms traditional methods and shallow models, particularly in challenging scenarios with low SNR and large sample sizes. Key conclusions from our results include:

- Traditional linear models like LR and SVM are inadequate for PEA, as they struggle to capture the complex, non-linear gene interactions within pathways. This results in higher Type I error rates and lower statistical power, especially in large and noisy datasets.
- Traditional PEA methods such as GSEA and ORA perform well in simple scenarios with high SNR and small sample sizes but are highly sensitive to noise and show limited improvements with increased sample sizes. This limits their utility in large-scale, multi-center studies where noise may obscure subtle biological signals.
- Deep learning-based methods like PASNet and DeepHisCom offer better performance in terms of power and Type I error control than traditional methods, but they are still sensitive to changes in SNR or come with high computational costs.
- GPNet consistently exhibits superior performance, showing robustness to noise while effectively improving results as sample sizes increase. It offers a scalable and efficient solution for pathway enrichment in complex, multi-center datasets, providing accurate results with minimal Type I error and high power, even in low SNR conditions.

In this article, we validate the feasibility (as shown in Fig. 2) and computational efficiency (as shown in Table 1) of the proposed $P$-value computation method by combining it with four classification methods: GPNet, PASNet, LR, and SVM. However, it is important to note that the performance of this method is highly dependent on the performance of the classifier. The superior PEA results achieved by GPNet in this study are largely due to its stronger classification capabilities. The purpose of introducing this $P$-value computation method is to transform the PEA task into a well-studied classification problem, enabling future researchers to transfer knowledge from classification tasks to PEA tasks.

**Key Points**

- Developed a novel pathway analysis method using Gene PointNet (GPNet) with a new $P$-value computation approach based on the classification confusion matrix.
- Demonstrated superior performance in handling low signal-to-noise ratio (SNR) and large sample-size datasets compared to traditional methods like ORA and FCS.
- Benchmarked against traditional statistical methods, shallow machine learning models, and other deep learning techniques, showing significant improvements in Type I error reduction and power.
- Validated the method on both simulated and real-world RNA-Seq datasets (TCGA breast cancer), proving its robustness for large, multi-center studies.
- Eliminated the need for computationally expensive permutation tests, making pathway analysis more efficient without compromising accuracy.
